# Omega 3 fatty acid supplementation after myocardial infarction: a systematic review and meta-analysis

**DOI:** 10.1186/s12872-019-1086-3

**Published:** 2019-06-04

**Authors:** Federico Popoff, Giselle Balaciano, Ariel Bardach, Daniel Comandé, Vilma Irazola, Hugo Norberto Catalano, Ariel Izcovich

**Affiliations:** 1 Ministry of health, Buenos Aires, Argentina; 2 National Ministry of Health of Argentina, Hospital Alemán, Buenos Aires, Argentina; 3 Institute for Clinical Effectiveness and Health Policy, Buenos Aires, Argentina

**Keywords:** Omega 3 fatty acid, Polyunsaturated fatty acids, Myocardial infarction, Secondary prevention, Systematic review

## Abstract

**Background:**

The purpose of this review is to examine the effect of Omega-3 Fatty acids on mortality, morbidity, and adverse events in patients with acute myocardial infarction (AMI).

**Methods:**

Data Sources: MEDLINE, EMBASE, and the Cochrane Library through May 2018. Study Selection: Randomized Controlled trials (RCT). Certainty of evidence was assessed with the GRADE system. Interventions: omega 3 fatty acids against placebo or no treatment. Primary and secondary outcomes: All-cause death, cardiovascular death, new AMI, stroke, need for therapeutic angioplasty or By-pass, new diagnosis of cancer and incidence of adverse events.

**Results:**

For the efficacy endpoints we included 10 RCT (24,414 patients). Omega 3 fatty acids probably make little or no difference to all-cause mortality (4 studies 9141 patients RR 1.06 - CI95% 0.90 to 1.27, moderate certainty), cardiovascular mortality (3 studies 4304 patients RR 0.93 - CI95% 0.63 to 1.37, moderate certainty), new AMI (RR 1.24 CI95% 0.71 to 2.14 - moderate certainty), any cardiovascular event (RR 0.95 95%CI 0.86 to 1.05; low certainty due to risk of bias and imprecision), and stroke (RR 1.2 95%CCI 0,66–2,19 - moderate certainty). Regarding adverse events, we are uncertain if Omega 3 fatty acids improve/reduce non severe adverse events (RR 1.39 95% CI 0.36 to 5.34; very low certainty). There is probably little or no difference in the outcome suspension due to adverse events (RR 1.19 CI 95% 0.97 to 1.47; moderate certainty).

**Conclusions:**

For adult patients with AMI, omega 3 fatty-acids probably yield no benefit to patient important outcomes.

**Electronic supplementary material:**

The online version of this article (10.1186/s12872-019-1086-3) contains supplementary material, which is available to authorized users.

## Background

n-3PUFAs are a family of polyunsaturated fatty acids, named as such because of the positioning of the first double carbon bond on the third atom from the methyl end of the acyl chain. Dietary sources of Omega 3 include certain nuts and seeds, such as walnuts, flaxseed and rapeseed (canola) oil, fatty fish, some white fish, shellfish and other seafood such as seaweed, and certain eggs and animal products, depending on the animal’s diet.

Proposed benefits of omega 3 fatty acids include lowering of blood pressure, reducing serum triglyceride concentration, increasing plaque stability and improving endothelial function [1–4]. In the context of previous acute myocardial infarction the mentioned omega 3 fatty acids plaque stabilization properties could result in significant benefits [4–7].

Multiple randomized controlled trials (RCT) evaluating the efficacy and safety safety of fatty acids have been published and their results summarized in different systematic reviews. However those published analysis have important limitations as we describe in the Additional file 1: Appendix 1. Furthermore, none of them performed a differential AMI patients analysis. In this context, we consider that a new systematic review (SR) is justified. The present systematic review and meta-analysis aimed to improve estimations and to assess whether dietary or supplemental omega 3 fatty acids affects total or cardiovascular mortality in the context of myocardial infarction secondary prevention.

## Methods

This research is not a clinical trial and therefore does not need to be registered.

### Search strategy and elegibility criteria

We searched for randomized controlled trials comparing omega 3 fatty acids against placebo or no treatment in the following literature databases, regardless of publication status and without language restrictions: the Cochrane Central Register of Controlled Trials from the Cochrane Library, MEDLINE, EMBASE, Epistemonikos and LILACS from inception until May 2018.Details of the full search strategies are provided in the Additional file 1: Appendix 2. Our gray-literature search included searches in Grey Matters Tool [8]. We also searched the Canadian Agency for Drugs and Technology in Health, Google Scholar, Trip Database, National Institute for Health and Care Excellence, McMaster University, McMaster Health Forum, PROSPERO, ClinicalTrials.gov, and manually examined the reference lists of all reviews identified.

As for the inclusion criteria, we included RCTs of adults that suffered a myocardial infarction (according to the study’s definition) and were randomized to receive omega 3-fatty acid supplementation at doses greater than or equal to 400 mg daily versus placebo/No treatment. The treatment should have started within 6 weeks after the initial diagnosis of the myocardial infarction. We considered any mode of administration of the intervention, such as dietary supplementation (fish oils, soya bean oils, seeds, refined EPA, DHA, ALA) or, oil or capsule form or as foodstuffs. To be eligible, studies had to report at least one of the following outcomes: All-cause death, cardiovascular death, new acute myocardial infarction, stroke, need for therapeutic angioplasty or By-pass, new diagnosis of cancer and incidence of adverse events.

### Study selection and data extraction

Two investigators (G.B. and F.P.) independently reviewed the titles and abstracts identified and full texts of included articles in order to determine eligibility using the EROS tool for systematic reviews early phases [9]. Disagreements or uncertainties were resolved by consensus of the whole team with an additional investigator (A.I.). We accepted the primary authors’ definition of AMI, stroke, adverse event and serious adverse event.

The risk of bias was assessed independently by two reviewers on an outcome by outcome basis using a modification of the Cochrane Risk of Bias Tool which considers, sequence generation, allocation concealment, blinding, number of patients with missing outcome data, selective outcome reporting, and other sources of bias [10]. We used the Grading of Recommendations Assessment, Development and Evaluation system to assess the certainty of the effect (also known as quality of evidence or confidence in evidence) for each outcome and for the entire body of evidence [11, 12]. Certainty of the effect takes into consideration the study design (in this case, randomized clinical trials); risk of bias, precision, consistency, directness of the evidence; and the probability of publication bias [13].

### Statistical analysis

We analyzed the data using Review Manager, version 5.3 (Cochrane Collaboration). We used random-effects models for all analyses (Mantel–Haenszel risk ratios [RRs] for dichotomous outcomes) since significant heterogeneity was expected. Publication bias was assessed through visual inspection of funnel plots (Additional file 1: Appendix 3) and the subjective impression of the reviewers (G.B., F.P. and A.I.) considering the size of the included studies and sponsorship. We also contacted investigators to consult whether they had knowledge of other potentially relevant unpublished trials.

We used Cochrane’s test for heterogeneity to determine whether studies in a meta-analysis evaluated the same underlying sizes of effect. We used the I^2^ statistic to test the degree of heterogeneity among studies (the proportion of total observed variability due to genuine variation rather than random error within studies) [14].

We planned a priori the following subgroup analyses as possible explanations for heterogeneity: 1) type of fatty acid: eicosapentaenoic acid and docosahexaenoic acid versus alpha-linolenic acids with a postulated larger effect for the latter [9, 15]; 2) Dose effect: high dose (consumption of more than 4.5 g daily) of Omega 3 fatty acids could be associated with a larger treatment benefit; 3) Type of omega 3: synthetic omega 3 (in comparison with dietary recommendations with increased omega 3 fatty acid intake) could be associated with a larger treatment benefit; 4) Risk of bias: Studies with high risk of bias could be associated with a larger treatment benefit. We visually analyzed the results of each subgroup comparison and additionally tested for interaction by using a chi-square significance test [14].

### Dealing with missing data

For the primary analysis we used a complete case-analysis approach, i.e. we excluded participants considered to have missing data. For those outcomes in which a clinically significant effect was observed (Relative risk CI95% not including 1), we performed a sensitivity analysis to challenge the possibility of risk of bias due to missing data following the approach described by Guyatt et.al [16, 17] (complete description of the implemented sensitivity analysis is available in Additional file 1: Appendix 4).

### Patient and public involvement

Patients were not involved in this review.

## Results

### Study characteristics

**Fig. 1:**
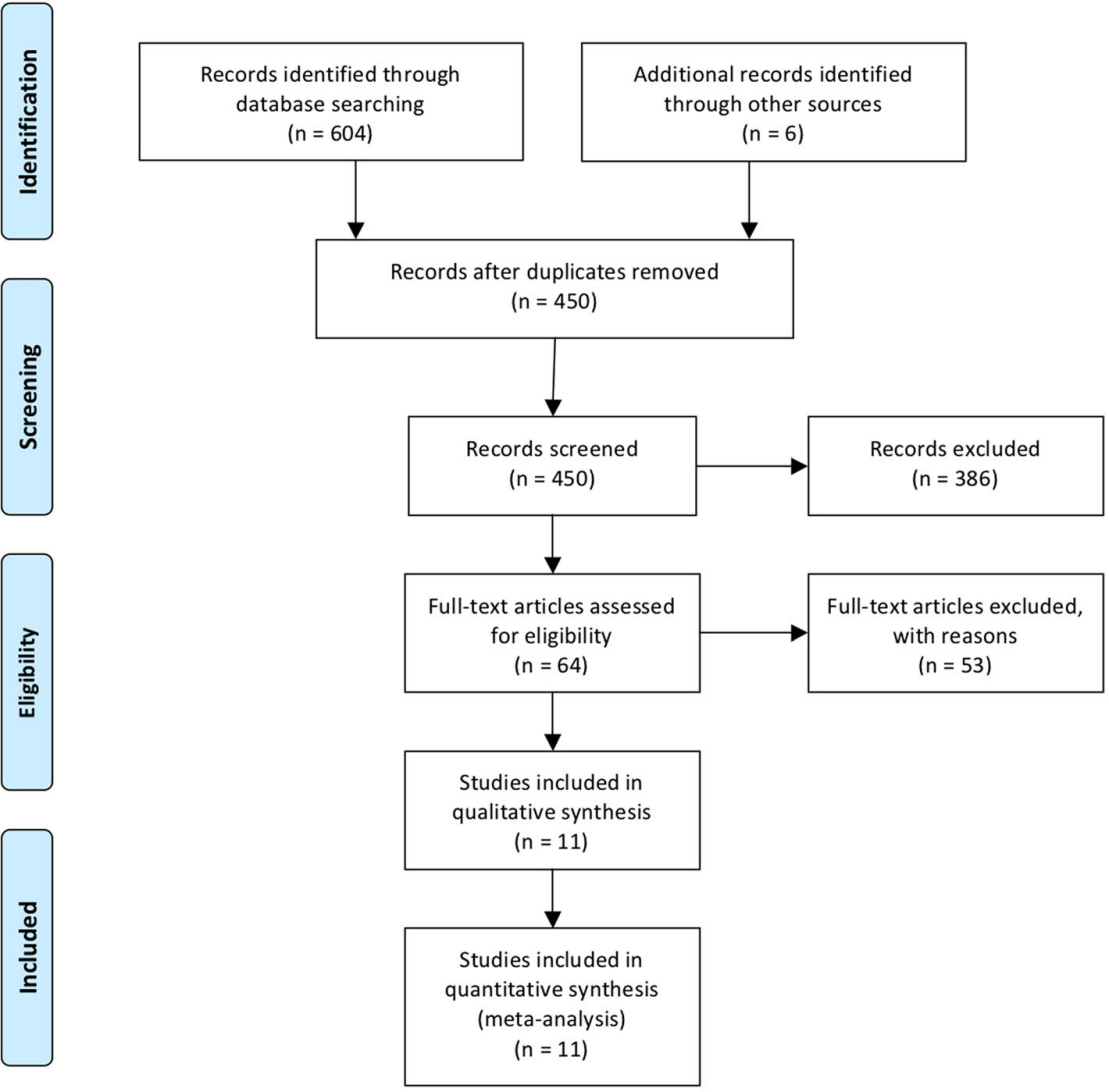
Flow chart of study selection

We identified 610 potentially relevant records. After screening titles, abstracts and full texts we included 11 publications for quantitative analysis (Fig. 1.).

The characteristics of the 11 RCTs included are summarized in Table 1. In six [18–23] of them the intervention was implemented in the form of dietary recommendations while EDH-EPA (synthetic form of omega 3 fatty acids) was implemented in the remaining four [24–28].

**Table 1:** Characteristics of included studies

Author, Year	Participants	Intervention	Comparison	Follow Up	Outcomes assesed	Funded by
Kromhout, D, 2010 [18]	4837 adults with AMI	Margarine with 400 mg of EPA–DHA, 2 g of ALA, or a combination of EPA–DHA and ALA	Placebo margarine	40 months	Major cardiovascular events, incident cardiovascular disease, death from cardiovascular disease, all-cause death, treatment suspension due to adverse events.	The Netherlands Heart Foundation, the National Institutes of Health, and Unilever R&D, the Netherlands.
de Lorgeril M, 1999 [19]	605 survivors of a first AMI	Mediterranean type of diet	No dietary advice	60 months	Cardiovascular death, all-cause mortality, nonfatal acute MI, unstable angina, stroke, heart failure, need for myocardial revascularization, need of therapeutic revascularization	CETIOM, ONIDOL, ASTRA-CALVE and the Fondation pour la Recherche Médicale
Marchioli, 2001 [24]	11,323 adults with recent (3 months) myocardial infarction	Gelatine capsules containing 850–882 mg (EPA) and (DHA)	No supplement	42 months	All-cause death, nonfatal myocardial infarction, nonfatal stroke, cardiovascular death, nonfatal MI, nonfatal stroke, cancer, need of therapeutic revascularization	Bristol-Myers Squibb, Pharmacia-Upjohn, Società Prodotti Antibiotici, and Pfizer. Pharmacia-Upjohn and Società Prodotti Antibiotici
Nilsen, D, 2001 [25]	300 adults, who had had an AMI	Capsules containing 850–882 mg (EPA) and (DHA)	Corn oil	24 months^a^	Cardiac death, resuscitation, recurrent MI, unstable angina, revascularization death from other causes	Pharmacia-Upjohn A/S and by Pronova A/S
Rauch, B, 2010 [26]	3851 adults within 3 to 14 days after acute myocardial infarction	Capsules containing 1 g omega-3 (460 mg EPA, 380 mg DHA)	Placebo	12 months	Cardiovascular death, total mortality, major adverse cerebrovascular, total mortality, reinfarction, stroke revascularization, cancer, treatment suspension due to adverse events, need of therapeutic revascularization	Trommsdorff GmbH & Co. KG Arzneimittel, and Pronova Biopharma
Singh, R, 1998 [27]	404 adults judged likely to have suffered AMI with onset of symptoms in the preceding 24 h	Capsules containing 180 mg EPA and 120 mg DHA	Placebo	12 months	Sudden cardiac death, angina pectoris, all-cause, total cardiac deaths, MI, nonfatal reinfarcation, total cardiac events	No clear founding reported.
Tuttle, 2008 [20]	100 adults recruited 6 weeks after first AMI	Mediterranean-style diet	No dietary advice	24 months	All-cause death, cardiac death, MI, heart failure, unstable angina pectoris, stroke	State Attorney General Vitamins Settlement Fund, The Heart Institute of Spokane and Providence Medical Research Center, Sacred Heart Medical Center and Deaconess Medical Center. Authors reported conflicts of interest.
Burr 1989 [21]	2033 male adults who had recovered from AMI	Dietary advice: at least two weekly portions (200–400 g) of fatty fish	No dietary advice	24 months	All-cause death, cardiac death, non-fatal MI, total cardiovascular events	The Welsh Heart Research Foundation, the Flora Project and the Health Promotion Research Trust for financial support.
Borchgrevink 1966 [22]	200 male adults with a diagnosis of AMI	10 ml. of linseed oil per day-50% linolenic acid, 17 linoleic acid, 19% oleic acid, and 14% saturated fatty acids.	Corn oil	10 months	All-cause death, cardiac death, non-fatal MI, total cardiovascular events	Nyegaard & Co
Morris 1968 [23]	393 male adults who had recovered from their first AMI	Dietary advice: soja bean oils	No dietary advice	60 months	Cardiac death, all-cause mortality, non-fatal MI	Medical Research Council
Heydari 2016 [28]	358 adults with AMI	4 one-gram capsules per day (EPA 465 mg and DHA, 375 mg)	Corn oil (600 mg linoleic acid, no O-3FA)	24 months	Left-Ventricle remodeling, All-cause mortality	The National Heart, Lung, and Blood Institute of the National Institutes of Health funded this study

*AMI* Acute myocardial infraction

^a^Grundt et al. [29] published in 2004 a long term follow up of the cohorts in Nilsen trial

### Risk-of-Bias assessment

**Fig. 2:**
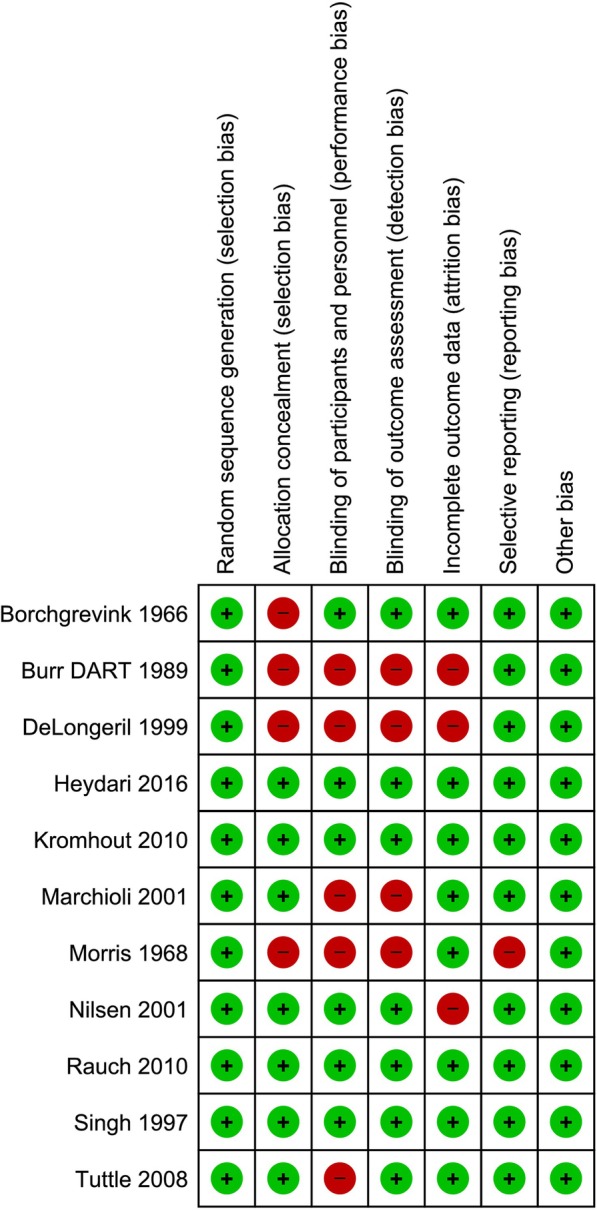
Risk-of-Bias of included studies

In five of the included studies [18, 22, 25–28], patients, investigators and outcome assessors were blinded. Four of those studies were judged as low risk of bias as no additional methodological issues were noted. Regarding the remaining studies, six were judged as to carry moderate or high risk of bias (Fig. 2). Although one of the included studies had no apparent methodological limitations, we decided to judge it as high risk of bias [27] because the author was accused of misconduct and data fabrication in two different trials in which he was involved [30, 31]. We assumed the trials to have important lost to follow-up when the authors did not offer enough information to analyze the impact of missing data or if the performed sensitivity analysis significantly altered the effect estimate or the confidence interval for each outcome. (Additional file 1: Appendix 4).

### All-cause mortality

**Fig. 3:**
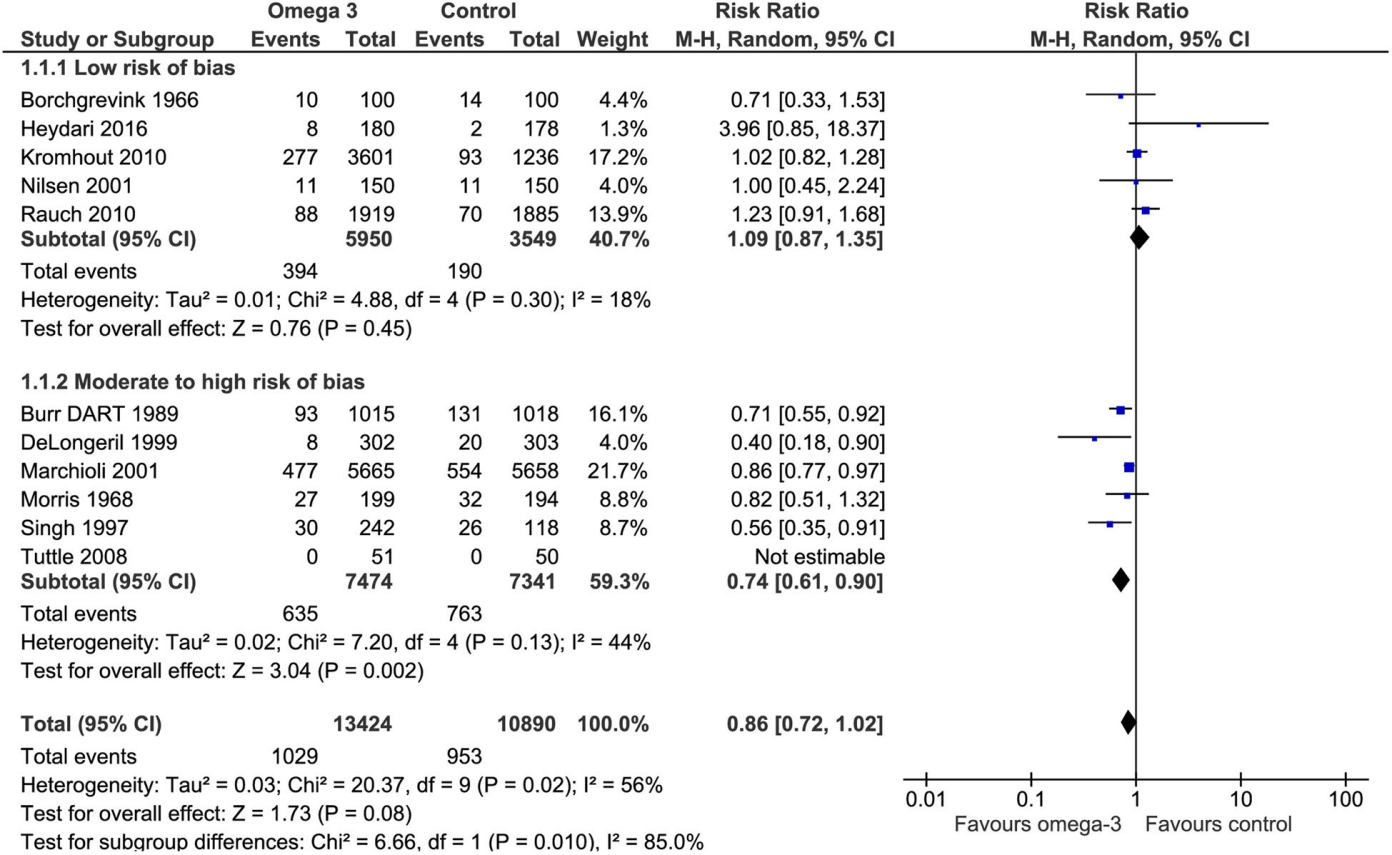
Forest plot of comparison: Omega 3 vs. Placebo, outcome: 1.1 All-cause mortality - Risk of bias subgroup

All the included trials addressed this outcome. Omega 3 fatty acids reduced the risk of all-cause mortality (RR 0.86, CI 95% 0.72 to 1.02). Considering the basal risk (risk without the intervention) as the *mean* of the risk in the control groups of the included RCT, the mortality reduction was 1.4% (CI95% 2.5 to 0) at a mean follow up of 3 years. We judged the certainty in the estimates of effect as low due to risk of bias, imprecision and inconsistency (I^2^ 85%) (Fig. 3), (Table 2).

**Table 2:** Summary of finding Table for Omega 3 for patients with myocardial infarction

Omega 3 compared to placebo for Patients with AMI							
		Patient or population: Patients with AMI Intervention: Omega 3 Comparison: Placebo					
Outcome № of participants (studies)	Relative effect (95% CI)	Anticipated relative effects (95% CI)	Anticipated absolute effects (95% CI)*			Certainty	What happens
			Without Omega 3 vs Placebo	With Omega 3 vs Placebo	Absolute Difference		
All-cause mortality follow up: mean 34 months № of participants: 24314 (11 RCTs)	RR 0.86 (0.72 to 1.02)	14% fewer (28% fewer to 2% more)	8.3%	7.1% (5.9 to 8.5)	0.9% fewer (2.4 fewer to 2.0 more)	⊕ ⊕ ◯◯ LOW^a,b,c,d^	Omega 3 fatty acids probably may make little or no difference to all-cause mortality
All-cause mortality - Risk of bias subgroup - Low risk of bias follow up: 26 months № of participants: 9949 (5 RCTs)	RR 1.09 (0.87 to 1.35)	9% more (13% fewer to 35% more)	8.3%	9% (7.2 to 11.2)	0.7% more (1.1 fewer to 2.9 more)	⊕ ⊕ ⊕◯ MODERATE^e^	Omega 3 fatty acids probably make little or no difference to all-cause mortality
Cardiovascular mortality follow up: 34 months № of participants: 19119 (9 RCTs)	RR 0.77 (0.65 to 0.91)	23% fewer (35% fewer to 9% fewer)	6.4%	4.9% (4.1 to 5.8)	1.5% fewer (2.3 fewer to 0.6 fewer)	⊕ ⊕ ◯◯ LOW^a,d,f^	Omega 3 fatty acids probably may reduce cardiovascular mortality
Cardiovascular mortality - Risk of bias subgroup - Low risk of bias follow up: 13 months № of participants: 11645 (3 RCTs)	RR 0.93 (0.63 to 1.37)	7% fewer (37% fewer to 37% more)	6.4%	6.0% (4.0 to 8.8)	0.4% fewer (2.4 fewer to 2.4 more)	⊕ ⊕ ⊕◯ MODERATE^e^	Omega 3 fatty acids probably make little or no difference to cardiovascular mortality
Acute myocardial infarction follow up: 41 months № of participants: 13282 (7 RCTs)	RR 0.77 (0.60 to 0.99)	23% fewer (40% fewer to 1% fewer)	9.6%	7.4% (5.7 to 9.5)	2.2% fewer (3.8 fewer to 0.1 fewer)	⊕ ⊕ ◯◯ LOW^f,g,h^	Omega 3 fatty acids may reduce acute myocardial infarction
Acute myocardial infarction - Low risk of bias subgroup follow up: 18 months № of participants: 6823 (2 RCTs)	RR 1.24 (0.71 to 2.14)	24% more (29% fewer to 14% more)	9.6%	11.8% (6.8 to 20.4)	2.3% more (2.8 fewer to 10.9 more)	⊕ ⊕ ⊕◯ MODERATE^e^	Omega 3 fatty acids probably make little or no effect on acute myocardial infarction
Ischaemic Stroke follow up: 39 months № of participants: 14262 (5 RCTs)	RR 1.20 (0.66 to 2.19)	20% more (34% fewer to 19% more)	1.2%	1.4% (0.8 to 2.6)	0.2% more (0.4 fewer to 1.4 more)	⊕ ⊕ ◯◯ LOW^e,i^	Omega 3 fatty acids probably may make little or no difference to stroke
Need of therapeutic revascularization follow up: 35 months № of participants: 15732 (3 RCTs)	RR 1.00 (0.91 to 1.10)	0.0% fewer (9% fewer to 10% more)	20.9%	20.9% (19.0 to 23.0)	0.0% fewer (1.9 fewer to 2.1 more)	⊕ ⊕ ◯◯ LOW^e,j^	Omega 3 fatty acids probably may make little or no difference to the need of revascularization
Treatment suspension due to adverse events follow up: 28 months № of participants: 8641 (2 RCTs)	RR 1.19 (0.97 to 1.47)	19% more (3% fewer to 47% more)	4.2%	5.0% (4.1 to 6.2)	0.8% more (0.1 fewer to 2.0 more)	⊕ ⊕ ⊕◯ MODERATE^e^	Omega 3 fatty acids probably make little or no difference to suspension due to adverse events
Cancer follow up: 33 months № of participants: 15127 (2 RCTs)^k^	OR 1.25 (0.94 to 1.66)	25% more (6% fewer to 66% more)	1.2%	1.4% (1.1 to 1.9)	0.3% more (0.1 fewer to 0.7 more)	⊕◯◯◯ VERY LOW^e,m^	Omega 3 fatty acids probably make little or no difference to suspension due to cancer

^*^The risk in the intervention group (and its 95% confidence interval) is based on the assumed risk in the comparison group and the relative effect of the intervention (and its 95% CI). *CI* Confidenceinterval, *RR* Risk ratio, *OR* Odds ratio

GRADE Working Group grades of evidence

High certainty: We are very confident that the true effect lies close to that of the estimate of the effect

Moderate certainty: We are moderately confident in the effect estimate: The true effect is likely to be close to the estimate of the effect, but there is a possibility that it is substantially different

Low certainty: Our confidence in the effect estimate is limited: The true effect may be substantially different from the estimate of the effect

Very low certainty: We have very little confidence in the effect estimate: The true effect is likely to be substantially different from the estimate of effect

Explanations

^a^The effect estimate was obtained from trial including six evaluated to have high risk of bias (Burr, De Longeril, Marchioli, Morris, Singh, Tuttle)

^b^The apparent benefit for this outcome was influenced by the effect of trials that met our criteria for moderate to high risk of bias. As there was no statistical heterogeneity the certainty was not downgraded a whole point

^c^The CI 95% crosses the no effect line (1), including the possibility of benefits and harms. As the 95% CI does not showpossible harms the certainty was not downgraded a whole point (see explanation b). Considering the posibility of drawbacks a thereshold below the no effect line could be set and therefore assuming a lack of benefit on the intervention there could be considered that there should not be downgraded for imprecision. As this thereshold should be set by a guideline pannelpannel we conidered the information to be imprecise

^d^It is possible to judge that is risk of publication bias for the moderate to high risk of bias soubgroup (small trials failing to prove effect of the intervention). We did not judge that this analisys would justify downgrading for risk of puclication bias

^e^The CI 95% crosses the no effect line (1), including the possibility of benefits and harms. Considering the posibility of drawbacks a thereshold below the no effect line could be set and therefore assuming a lack of benefit on the intervention there could be considered that there should not be downgraded for imprecision. As this thereshold should be set by a guideline pannelpannel we conidered the information to be imprecise

^f^The apparent benefit for this outcome was influenced by the effect of trials that met our criteria for moderate to high risk of bias

^g^The effect estimate was obtained from trial including five evaluated to have high risk of bias (De Longeril, Marchioli, Morris, Singh, Tuttle)

^h^As the 95% CI does not show possible harms the certainty was not downgraded a whole point (see explanation b). Considering the posibility of drawbacks a thereshold below the no effect line could be set and therefore assuming a lack of benefit on the intervention there could be considered that there should not be downgraded for imprecision. As this thereshold should be set by a guideline pannelpannel we conidered the information to be imprecise

^i^The effect estimate was obtained from trial including four evaluated to have high risk of bias (Burr, De Longeril, Marchioli, Tuttle)

^j^The effect estimate was obtained from trial including two evaluated to have high risk of bias (De Longeril, Marchioli)

^k^The follow up was to short to evaluate cancer incidence. The diagnostic procedures to evaluate the incidence of cancer were not clearly described

### Cardiovascular mortality

**Fig. 4:**
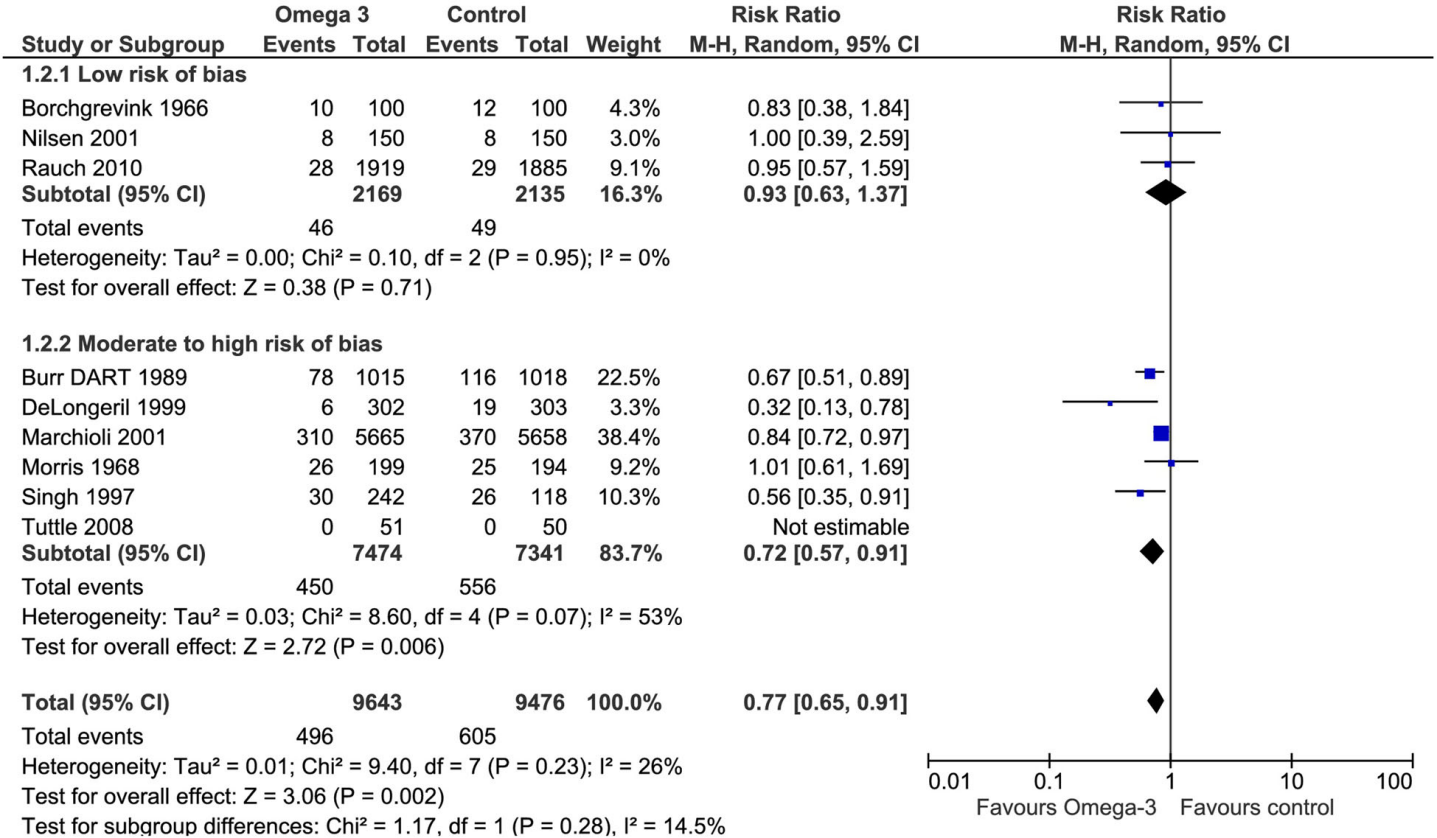
Forest plot of comparison: Omega 3 vs. Placebo, outcome: 1.2 Cardiovascular mortality - Risk of bias subgroup

Nine of the included trials addressed this outcome. Omega 3 fatty acids reduced the risk of cardiovascular mortality (RR 0.77, CI 95% 0.65 to 0.91). Considering the basal risk as the *mean* of the risk in the control groups of the included RCT, the cardiovascular mortality reduction was 1.5% (CI95% 2.3 to 0.6) at a mean follow up of approximately 3 years. We judged the certainty in the estimates of effect as low due to risk of bias and inconsistency (Fig. 4), (Table 2).

### Acute myocardial infarction

**Fig. 5:**
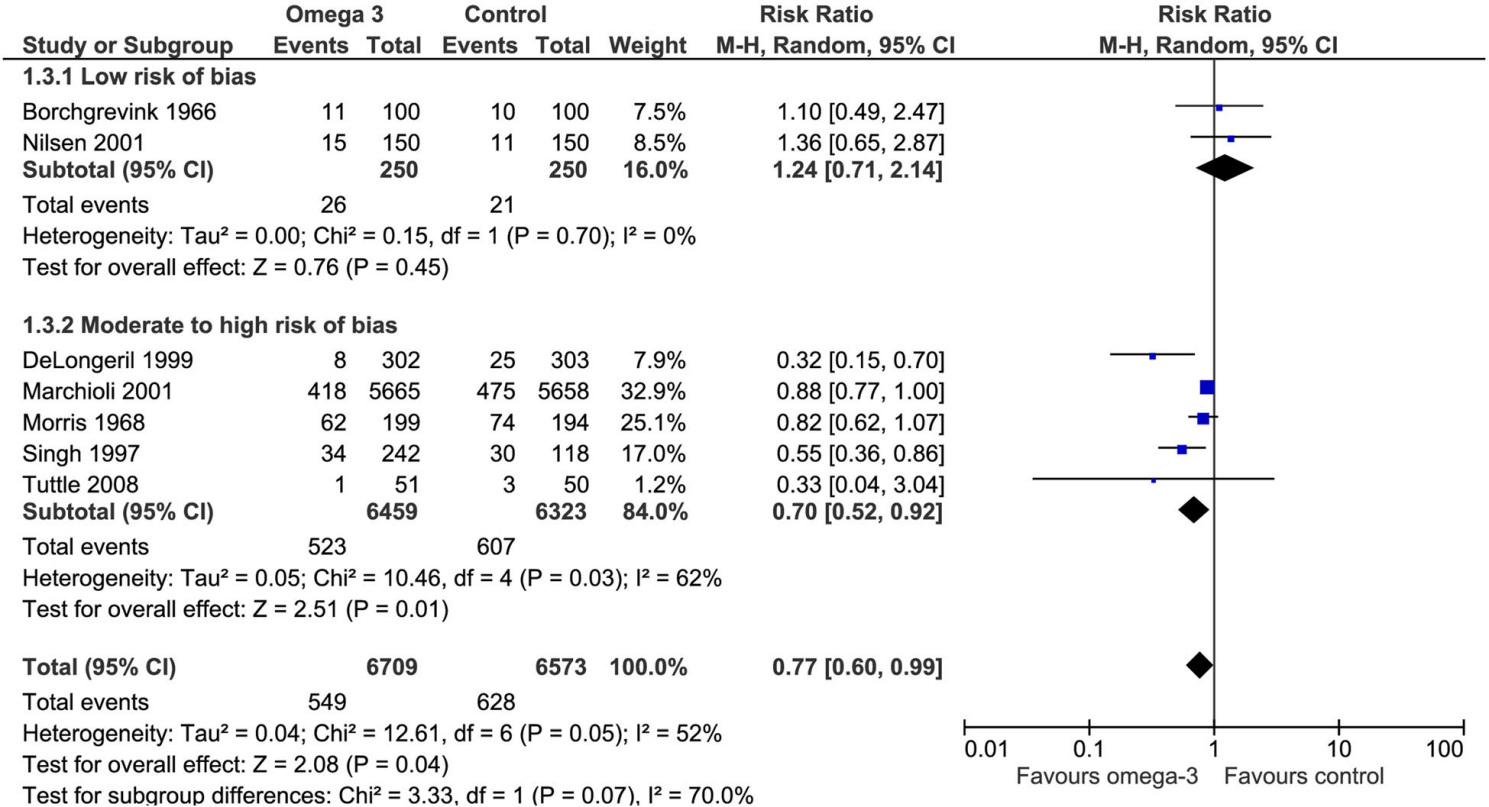
Forest plot of comparison: Omega 3 vs. Placebo, outcome: 1.3 Acute myocardial infarction- Risk of bias subgroup

Seven of the included trials addressed this outcome. Omega 3 fatty acids reduced the risk of Myocardial infarction (RR 0.77, CI 95% 0.6 to 0.99). Considering the basal risk as the *mean* of the risk in the control groups of the included RCT, the cardiovascular mortality reduction was 2.2% (CI95% 3.8 to 0.1) at a mean follow up of approximately 3 years. We judged the certainty in the estimates of effect as low due to risk of bias, imprecision and inconsistency) (Fig. 5), (Table 2).

### Stroke

**Fig. 6:**
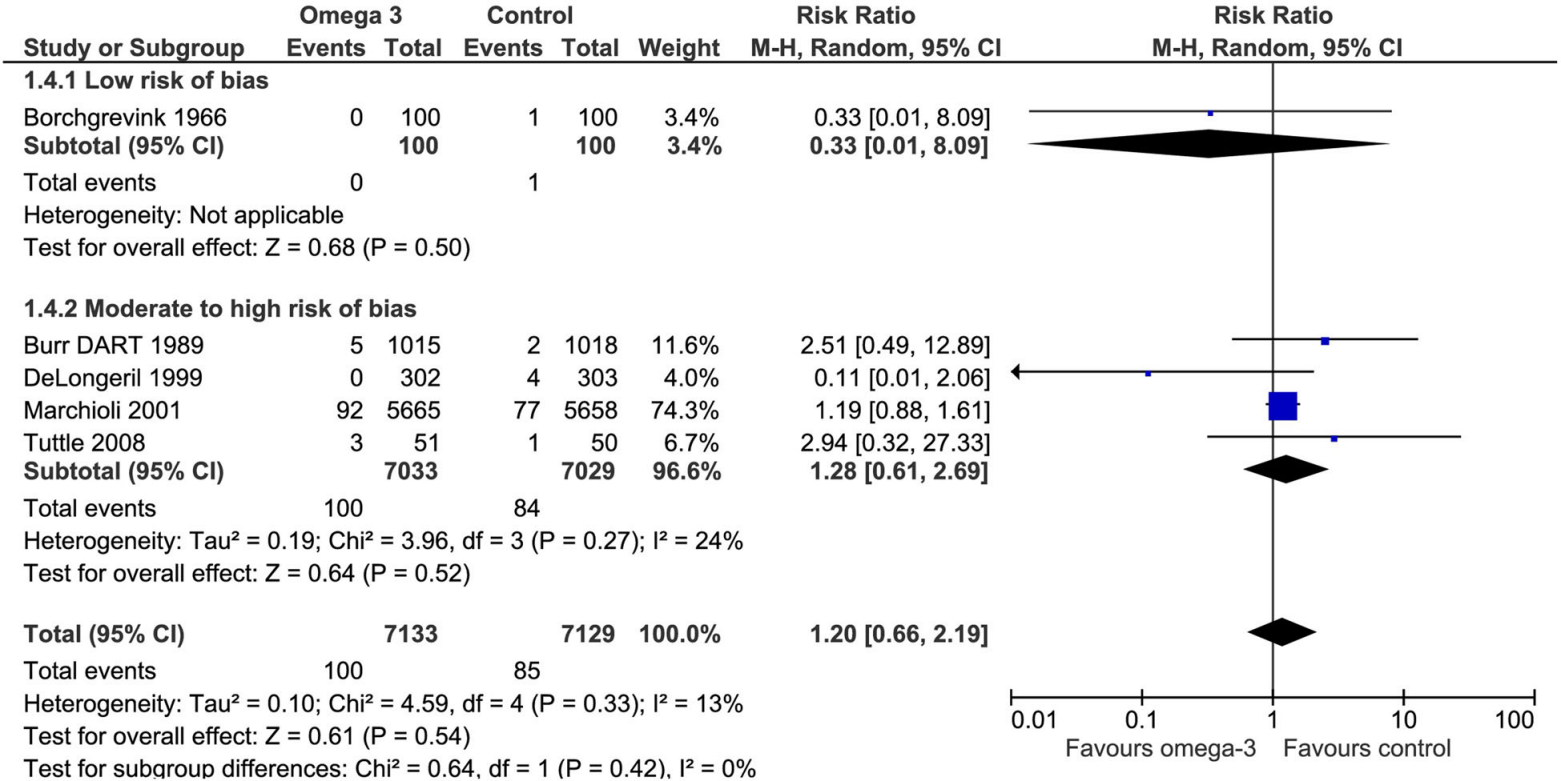
Forest plot of comparison: Omega 3 vs. Placebo, outcome: 1.4 Stroke - Risk of bias subgroup

Five of the included trials addressed this outcome. Omega 3 fatty acids did not reduce the risk of stroke (RR 1.2, CI 95% 0.66 to 2.19). Considering the basal risk as the *mean* of the risk in the control groups of the included RCT a marginal increase in the risk of stroke was observed RD 0.2% (CI95% -0.4 to 1.4%) at a mean follow up of approximately 3 years. We judged the certainty in the estimates of effect as moderate due to imprecision (Fig. 6), (Table 2).

### Need to therapeutic revascularization

**Fig. 7:**
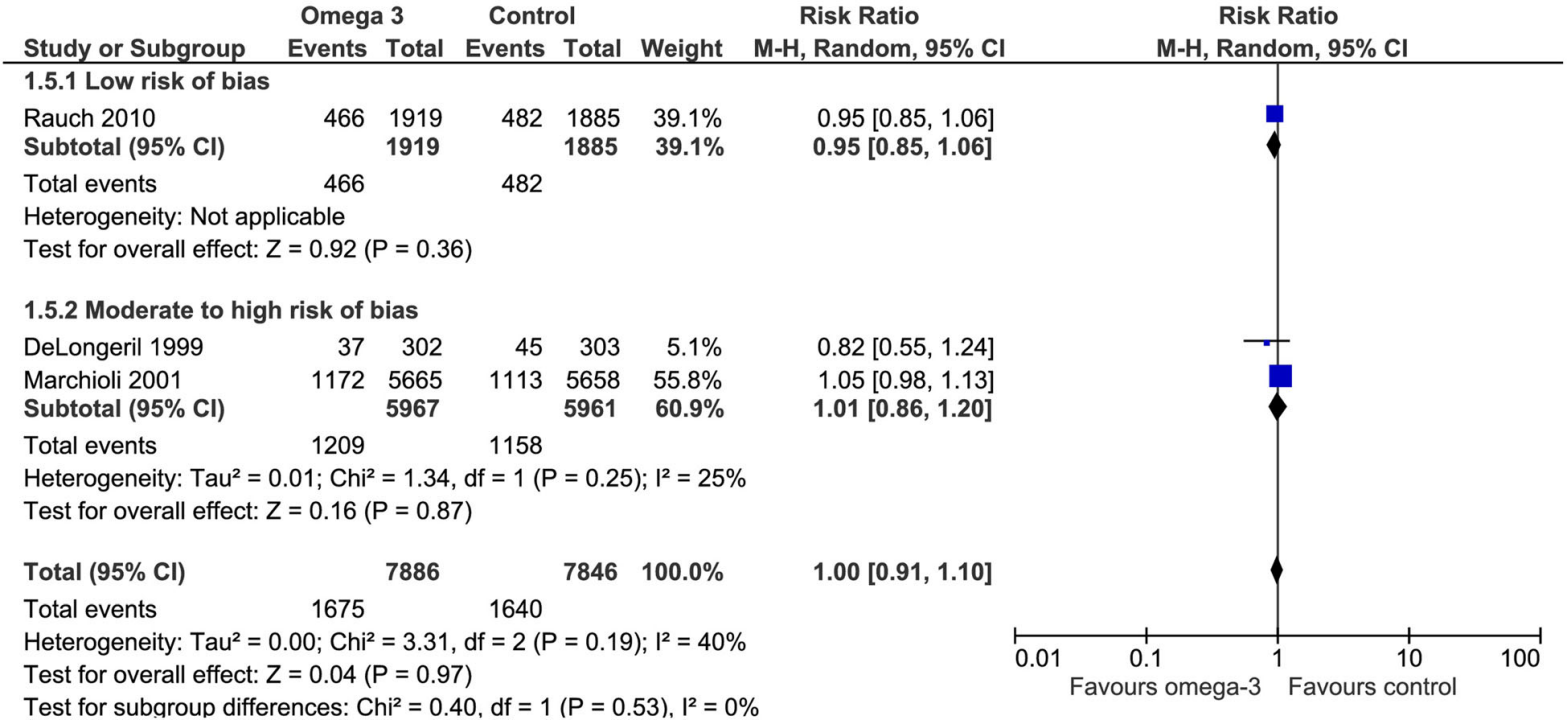
Forest plot of comparison: Omega 3 vs. Placebo, outcome: 1.5 Need to revascularization - Risk of bias subgroup

Three of the included trials addressed this outcome. Omega 3 fatty acids did not reduce the risk of therapeutic revascularization (RR 1.0 CI 95% 0.91). Considering the basal risk as the *mean* of the risk in the control groups of the included RCT no differences in the need of therapeutic revascularization were observed RD 0% (CI95% -1.9 to 2.4%) at a mean follow up of approximately 3 years. We judged the certainty in the estimates of effect as moderate due to imprecision (Fig. 7), (Table 2).

### Treatment suspension due to adverse events

**Fig. 8:**
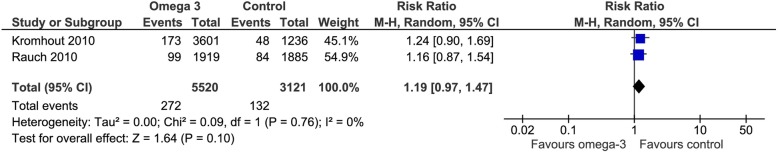
Forest plot of comparison: Treatment suspension due to adverse events

Two of the included trials addressed this outcome. Omega 3 fatty acids increased the relative risk of treatment suspension due to adverse effects (RR 1.19 CI 95% 0.97 to 1.47). Considering the basal risk as the *mean* of the risk in the control groups of the included RCT no differences in the risk of treatment suspension due to adverse effects was observed RD 0.3% (CI95% -0.1 to 0.7%) at a mean follow up of approximately 3 years. We judged the certainty in the estimates of effect as moderate due to imprecision (Fig. 8), (Table 2).

### Cancer

**Fig. 9:**
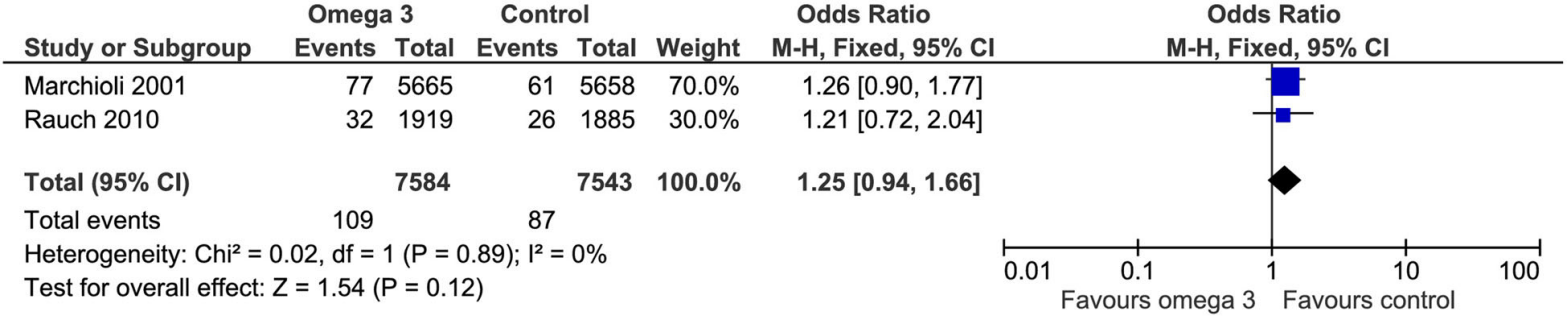
Forest plot of comparison: Cancer

Two of the included trials addressed this outcome. Omega 3 fatty acids increased the relative risk of cancer (RR 1.25 CI 95% 0.94 to 1.66). Considering the basal risk as the *mean* of the risk in the control groups of the included RCT only a marginal increase in cancer occurrence was observed RD 0.3% (CI95% -0.1 to 0.7%) at a mean follow up of approximately 3 years. We judged the certainty in the estimates of effect as very low due to imprecision and indirectness (Fig. 9). The results are shown in a Summary of Finding Table (Table 2).

### Subgroup analysis

Inconsistency was observed for overall mortality, cardiovascular mortality and myocardial infarction outcomes. In performing the prespecified risk of bias subgroup analysis we observed that the benefits (mortality, cardiovascular mortality and MI reduction), suggested by the overall pooled results, were not present when pooling the subgroup of studies in which the intervention was applied in a blinded fashion (low risk of bias). Although the test for subgroup differences was only statistically significant for overall mortality (*p* < 0.05) visual inspection of the forest plots suggest a similar subgroup effect for the three outcomes. The Fatty acid type subgroup analysis also showed a possible differential effect (less cardiovascular mortality) when EPA-DHA was implemented in comparison to ALA (RR 0.66, IC95% 0.35–1.27 for ALA vs. RR 0.82, IC95% 0.72–094) for EPA + DHA. No significant differences were observed when different doses were used.

We then decided to include, in the summary of findings tables, both the results of the overall pooled estimates and the results of the blinded trials pooled estimates (Table 2). We used primarily a 4 mg threshold to evaluate the effect of the dose of omega 3 fatty acid which showed no effect on the evaluated outcomes. A secondary analysis using a 1 mg threshold showed also no impact on those outcomes.

## Discussion

This systematic review provides moderate quality of evidence that Omega 3 fatty acids do not significantly reduce mortality or major vascular events in patients with acute myocardial infarction. Although the overall estimates of effects analysis suggested a reduction in mortality and AMI recurrence, based on the results of the subgroup analysis we consider that this finding is probably biased. Hence our main conclusions are based in the pooled estimates provided by the blinded RCT (low risk of bias subgroup). Nevertheless, we decided to report both estimates (overall and low risk of bias subgroup) in order to supply decision makers with all the information (see Table 2). Regarding adverse events, particular concern has been raised by the possibility that the intervention could cause cancer. This hypothesis was drawn based on the observation that omega-3 fats capsules could contain high levels of various toxic compounds such as mercury, polychlorinated biphenyl and dioxins [32–37]. Our results provide very low quality of evidence suggesting that omega 3 may increase the risk of cancer but the scarce number of events and the limited time of follow up make it difficult to draw definite conclusions regarding this outcome. This information, consistent with the conclusions of other systematic reviews on the topic [38–40], can help decision makers by supporting the fact that the risk of significant negative effects related to omega 3 fatty acids cannot be ruled out.

Even though we observed a possible subgroup effect in favor of DHA-EPA fatty acids, this is mainly based in differences between studies, as opposed to differences in subgroups within studies, and the differential effect was mainly influenced by moderate/high risk of bias trials. We consider that a true subgroup effect under these conditions is unlikely.

Our review has limitations,. Although we performed a thorough evidence search, we did not explored conference abstracts. Besides we did not include studies that randomized patients with AMI combined with other subpopulations (i.e patients with stroke) [41] as we could not gain access to individual patients’ data or AMI subgroup results for any of those identified trials [42, 43].

Although we understand that we could have missed relevant information as a consequence of the mentioned limitations, we consider that improbable. Our systematic review has also particular strengths. First, it provides the most comprehensive and trustworthy body of evidence up to date, including studies that were not included in other recent prior [19, 20, 22, 23, 28] reviews. While the conclusions of our systematic review in terms of the effects of the intervention are not different from the conclusions of some of the published reviews addressing similar questions, we believe that the analysis of the certainty of the evidence and the way in which we presented the results (following the GRADE approach) better reflects the trustworthiness of the information available, particularly regarding the absence of benefits in terms of mortality reduction and AMI recurrence.

As mentioned in the introduction, none of the published reviews on the topic, particularly the recently published Cochrane review [44], performed a complete and in-depth analysis of the effects of Omega 3 fatty acids on the population of patients with acute myocardial infarction. The most relevant differences of those SR with ours comprise: 1) None included all the available evidence [32, 44–46]; 2) Most did not perform a subgroup analysis considering the risk of bias of the included studies, which we believe, that in this particular scenario, is crucial to interpret the whole body of evidence [33, 46]; 3) Most included patients with cardiovascular risk factors, stable angina pectoris or other cardiovascular conditions but without previous events; whereas we focused our question on the group of patients with acute myocardial infarction hypothesizing that the Ingestion of omega-3 PUFAs including EPA and DHA may result in more significant benefits by attenuating the inflammatory response triggered by the myocardial injury [33, 44, 45].

The results and conclusions of those published reviews are inconsistent. While some report a positive effect of omega 3 fatty acids and even recommend its use [47], others claim that there is not enough evidence about the benefits of the intervention [44, 45]. One of the most recent reviews, published by Aung et al. [48], deserves a detailed description. Although the authors appropriately analyzed the results considering the risk of bias of the primary studies, they failed to include most of the evidence related to patients with previous MI (9 of 11 studies) [19–27], see Additional file 1: Appendix 1). The authors concluded that omega-3 fatty acids had no significant association with fatal or nonfatal coronary heart disease or any major vascular events. Similar considerations can be made regarding the Cochrane review [44]. Our results strengthen the ones seen in the reviews of Cochrane and Aung by expanding their findings to the high-risk subgroup of patients with previous MI.

## Conclusion

Omega 3 fatty acid supplementation probably yields no benefit to patient important outcomes for individuals with previous AMI. The results of our systematic review would provide useful information to panels aiming to elaborate recommendations for the management of patients with previous AMI.

### Strengths and limitations of this study

- The present systematic review provides estimations regarding the efficacy of supplemental omega 3 fatty acids in the context of myocardial infarction secondary prevention paying special attention to the risk of bias of the included studies
- Unlike most of the published reviews we focused in the population of patients that had suffered an acute myocardial infarction
- It provides the most comprehensive and trustworthy body of evidence up to date, including studies not included in any other published reviews [19, 20, 22, 23, 28].
- We performed a thorough analysis of the information and identified significant differences in the results of the primary studies that could be explained by methodological limitations in some of them.

## Additional file

Additional file 1: **Appendix 1.** Recently published relevant systematic reviews evaluating the intervention. **Appendix 2.** Details of the full search strategies. **Appendix 3.** Funnel plot of the All-cause mortality comparison. **Appendix 4.** Missing Outcome Data – Sensitivity analyses. (DOCX 106 kb)

## Abbreviations

ALA: Alpha-linolenic acids
AMI: Acute myocardial infarction
CI: Confidence interval
DHA: Docosahexaenoic acid
DPA: Docosapentaenoic acid
EPA: Eicosapentaenoic acid
GRADE: Grading of recommendations assessment, development and evaluation
PUFA: Poly-Insaturated fatty acids
RCT: Randomized controlled trials
RD: Risk difference
RR: Relative risk
RRD: Relative risk difference
SR: Systematic review
