# Supplementary material for: Omega 3 fatty acid supplementation after myocardial infarction: a systematic review and meta-analysis
Source: BMC Cardiovasc Disord. 2019 Jun 4;19:136. doi: 10.1186/s12872-019-1086-3 (PMC6549284; doi:10.1186/s12872-019-1086-3)
Supplement: Supplementary file 1 — Appendix 1. Recently published relevant systematic reviews evaluating the intervention. Appendix 2. Details of the full search strategies. Appendix 3. Funnel plot of the All-cause mortality comparison. Appendix 4. Missing Outcome Data – Sensitivity analyses. (DOCX 106 kb) [file 12872_2019_1086_MOESM1_ESM.docx]

Additional file

Additional file1: **Appendix 1**: Recently published relevant systematic reviews evaluating the intervention

|  | **Kwak 2012[8]** | **NICE 2013[10]** | **Hooper 2004[7]** | **Aung 2018 [9]** |
| --- | --- | --- | --- | --- |
| **All patient important Outcome assesment** |  |  |  |  |
| **Incuded all the availiable evidence** | 1 | 1 | 1 | 1 |
| **Adecuate lost to follow up assesment** | 2 | 2 | 2 | 2 |
| **Transparent evaluation of the quality of evidence** | 3 |  |  | 3 |

1 Failed to include at least one low risk of bias trial

2 No attempts were made to evaluate the potential impact of lost to follow up in individual trials in the effect estimate (through sensitivity analysis)

3 Failed to express absolute risk reduction of the intervention

Additional file 1**:** Appendix 2: Details of the full search strategies

| Database | Key-Words |
| --- | --- |
| Medline | (Fatty Acids, Omega-3[Mesh] OR omega 3[tiab] OR n-3 Fatty[tiab] OR n-3 PUFA[tiab] OR n 3 Polyunsatura*[tiab] OR Linolen*[tiab] OR Docosahexaenoic[tiab] OR Docosahexaenoate[tiab] OR Eicosapentanoic[tiab] OR Timnodonic[tiab] OR Icosapentaenoic[tiab]) AND (Myocardial Infarction[Mesh] OR Myocardial Infarct*[tiab] OR Stunned Myocard*[tiab] OR Myocardial Hibernat*[tiab] OR Cardiogenic Shock[tiab]) AND ((randomized controlled trial [pt] OR controlled clinical trial [pt] OR randomized controlled trials [mh] OR random allocation [mh] OR double-blind method [mh] OR single-blind method [mh] OR clinical trial [pt] OR clinical trials [mh]) OR ("clinical trial" [tw]) OR ((singl* [tw] OR doubl* [tw] OR trebl* [tw] OR tripl* [tw]) AND (mask* [tw] OR blind* [tw])) OR (placebos [mh] OR placebo* [tw] OR random* [tw] OR research design [mh:noexp]) NOT (animals [mh] NOT human [mh])) |
| LILACS | MH Ácidos Grasos Omega-3 OR Omega-3 OR n-3 Fatty OR n-3 PUFA OR n 3 Polyunsatura$ OR Linolen$ OR Docosahexaenoic$ OR Docosahexaenoa$ OR Dosahexenoat$ OR Eicosapentanoic$ OR Timnodonic$ OR Icosapentaenoic$ OR Grasos N-3 OR Graxos N-3) AND (MH Infarto del Miocardio OR Myocardial Infarct$ OR Infarto$ OR Myocard$ OR miocard$) [Palabras] |
| EMBASE | (Fatty Acids, Omega-3[Mesh] OR omega 3[tiab] OR n-3 Fatty[tiab] OR n-3 PUFA[tiab] OR n 3 Polyunsatura*[tiab] OR Linolen*[tiab] OR Docosahexaenoic[tiab] OR Docosahexaenoate[tiab] OR Eicosapentanoic[tiab] OR Timnodonic[tiab] OR Icosapentaenoic[tiab]) AND (Myocardial Infarction[Mesh] OR Myocardial Infarct*[tiab] OR Stunned Myocard*[tiab] OR Myocardial Hibernat*[tiab] OR Cardiogenic Shock[tiab]) |

List of contacted authors:

Dr Meyer - Double-Blind, Randomized, Controlled Trial of Fish Oil Supplements in Prevention of Recurrence of Stenosis After Coronary Angioplasty

Dr Sachs - Controlled trial of fish oil for regression of human coronary atherosclerosis

Dr Ness - Advice to Eat Fish and Mood: A Randomised Controlled Trial in Men with Angina

Dr Sarkkinen - Long term effects of three fat modified diets on serum lipids in free living hypercholesterolemic subjects

Dr Galan – SU.FOL.OM3 Trial: B-vitamins and N-3 polyunsaturated fatty acids supplementation and risk of recurrence of cardiovascular events

Dr Swahn - Omega-3 ethyl ester concentrate decreases total apolipoprotein CIII and increases antithrombin III in postmyocardial infarction patients

Dr Gajos - Effects of Polyunsaturated Omega-3 Fatty Acids on Responsiveness to Dual Antiplatelet Therapy in Patients Undergoing Percutaneous Coronary Intervention

Additional file 1: Appendix 3: Funnel plot of the All-cause mortality comparison


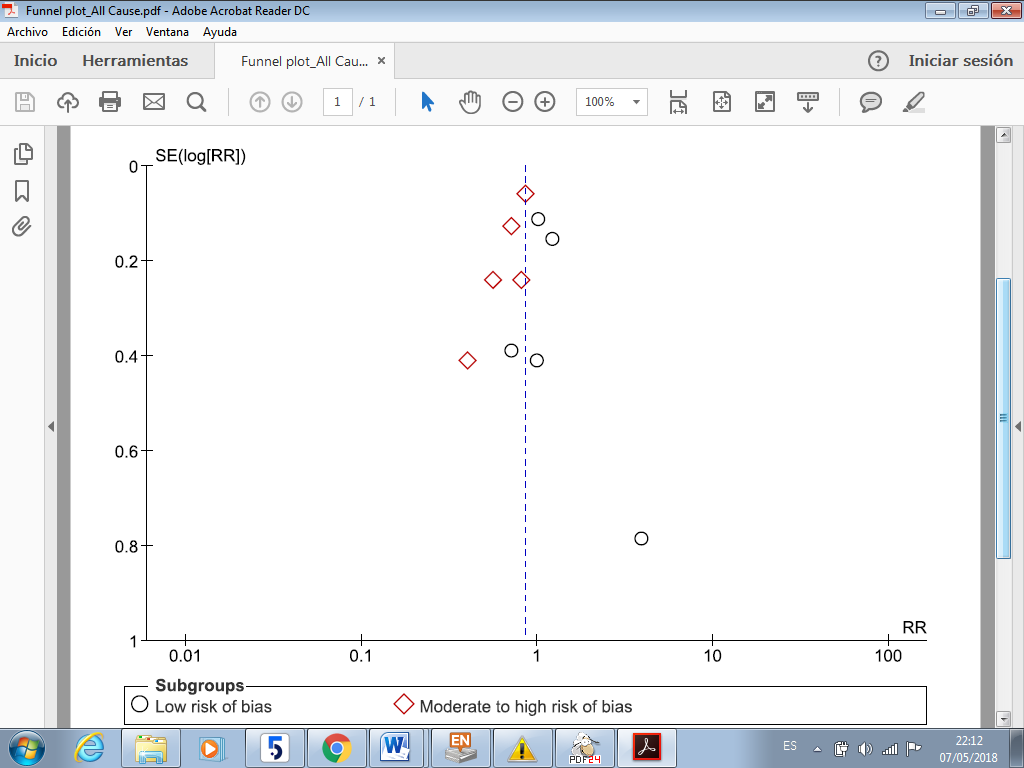


Additional file 1: Appendix 3 – Fig 1: Funnel plot of comparison: All-cause mortality. Considering the effect estimate for low (1,06) and moderate to high (0,74) risk of bias we assumed that there was no risk of bias, being possible to judge that is risk of bias for the moderate to high risk of bias group (small trials failing to prove effect of the intervention)


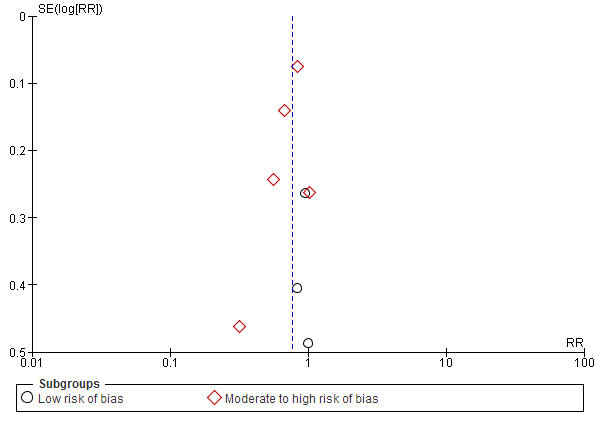


Additional file 1: Appendix 3 – Fig 2: Funnel plot of comparison: Cardiovascular mortality. See comment in Fig 1

Additional file 1: Appendix 4: Missing Outcome Data – Sensitivity analyses

To explore the effect of missing outcome data, we collected information on all missing outcome data from the included studies, and compared our primary complete case approach to a series of sensitivity analyses. For the sensitivity analyses, we assumed that the event rate was the same among control participants for whom data were missing and among those who were successfully followed. For the omega 3 groups, we calculated effects by using assumed ratios of event rate in persons with missing data compared with those who were successfully followed: 1.5:1, 2:1, 3:1, and 4:1 [19, 42]. We then determined whether the results withstood the range of assumptions, including the worst plausible assumption (4:1).
